# Identification of Breast Cancer Stem Cell Related Genes Using Functional Cellular Assays Combined With Single-Cell RNA Sequencing in MDA-MB-231 Cells

**DOI:** 10.3389/fgene.2019.00500

**Published:** 2019-05-22

**Authors:** Emma Jonasson, Salim Ghannoum, Emma Persson, Joakim Karlsson, Thomas Kroneis, Erik Larsson, Göran Landberg, Anders Ståhlberg

**Affiliations:** ^1^Department of Pathology and Genetics, Institute of Biomedicine, Sahlgrenska Cancer Center, Sahlgrenska Academy at University of Gothenburg, Gothenburg, Sweden; ^2^Department of Medical Biochemistry and Cell Biology, Institute of Biomedicine, Sahlgrenska Academy at University of Gothenburg, Gothenburg, Sweden; ^3^Department of Cell Biology, Histology and Embryology, Gottfried Schatz Research Center, Medical University of Graz, Graz, Austria; ^4^Department of Clinical Pathology, Sahlgrenska University Hospital, Gothenburg, Sweden; ^5^Department of Clinical Genetics and Genomics, Sahlgrenska University Hospital, Gothenburg, Sweden; ^6^Wallenberg Centre for Molecular and Translational Medicine, University of Gothenburg, Gothenburg, Sweden

**Keywords:** breast cancer, cancer stem cell, cell proliferation assay, mammosphere assay, single-cell analysis, single-cell RNA sequencing

## Abstract

Breast cancer tumors display different cellular phenotypes. A growing body of evidence points toward a population of cancer stem cells (CSCs) that is important for metastasis and treatment resistance, although the characteristics of these cells are incomplete. We used mammosphere formation assay and label-retention assay as functional cellular approaches to enrich for cells with different degree of CSC properties in the breast cancer cell line MDA-MB-231 and performed single-cell RNA sequencing. We clustered the cells based on their gene expression profiles and identified three subpopulations, including a CSC-like population. The cell clustering into these subpopulations overlapped with the cellular enrichment approach applied. To molecularly define these groups, we identified genes differentially expressed between the three subpopulations which could be matched to enriched gene sets. We also investigated the transition process from CSC-like cells into more differentiated cell states. In the CSC population we found 14 significantly upregulated genes. Some of these potential breast CSC markers are associated to reported stem cell properties and clinical survival data, but further experimental validation is needed to confirm their cellular functions. Detailed characterization of CSCs improve our understanding of mechanisms for tumor progression and contribute to the identification of new treatment targets.

## Introduction

Breast cancer is the most common cancer type affecting women worldwide and is one of the main causes of cancer-related deaths in women (Torre et al., 2015). It is a complex disease with many subtypes differing in prognosis and treatment options. Currently, breast cancer can be divided into four intrinsic subtypes; Luminal A, Luminal B, HER2-enriched and basal-like. The subtypesare further subgrouped based on their expression pattern of proliferation marker Ki67, HER2, and the hormone receptors ER (estrogen receptor) and PgR (progesterone receptor) (Reis-Filho and Pusztai, 2011; Senkus et al., 2013). Breast cancer displays both large inter- and intra-tumor heterogeneity, where each tumor contains small subpopulation of cancer stem cells (CSCs) that drive tumor progression. Similarly to normal stem cells, CSCs are able to self-renew and can give rise to progenitor cells as well as more differentiated cells (Reya et al., 2001; Beck and Blanpain, 2013; Kreso and Dick, 2014). It is also known that CSCs are important for metastasis and therapy resistance in breast cancer (Al-Hajj et al., 2003; Bhola et al., 2013; Lawson et al., 2015). Hence, detailed understanding of CSCs is important when developing novel treatment strategies.

Breast CSCs display several cellular features that can be used to enrich for this specific subpopulation. CSCs, in contrast to other tumor cells, display the ability to initiate tumor formation in immunodeficient mice (Al-Hajj et al., 2003). CSCs are also connected to therapy-resistance (Fillmore and Kuperwasser, 2008). Another property of CSCs is their ability to proliferate and differentiate under non-adherent cell culture conditions, which is used in the mammosphere formation assay (Dontu et al., 2003). Studies have also shown that CSCs, similarly to normal stem cells, often have a slow-dividing, and sometimes quiescent, phenotype (Dembinski and Krauss, 2009; Pece et al., 2010; Lyle and Moore, 2011). This cellular feature can be assessed with label-retention assays, where the ability of cells to maintain high amounts of an incorporated dye indicates few cell divisions (Lyle and Moore, 2011). Specific expression of cell surface markers, like CD44^+^/CD24^-/low^ (Al-Hajj et al., 2003), and activity of aldehyde dehydrogenase (Ginestier et al., 2007) are also useful when enriching for CSCs. However, all these features are not unique to CSCs but shared by other cells too, limiting their use.

Most performed breast CSC studies are on populations of cells that cannot reveal any information about CSC heterogeneity. Single-cell analysis overcomes this obstacle. In breast cancer, single-cell gene expression analysis have been used to study, for example, metastatic potential (Lawson et al., 2015; Nguyen et al., 2016), drug response (Lee et al., 2014; Savage et al., 2017), intratumoral heterogeneity (Chung et al., 2017), and cell state transitions (Akrap et al., 2016).

In this study, we first enriched for breast CSCs by collecting slow/non-dividing MDA-MB-231 cells, identified by a label-retention assay, that formed mammospheres in non-adherent cell culture conditions. We then performed single-cell RNA sequencing and used single-cell algorithms to define CSC subpopulations. We also studied CSCs transition into more differentiated cells and identified potential breast CSC-specific biomarkers.

## Materials and Methods

### Cell Culture

MDA-MB-231 luciferase cells were cultured in DMEM medium (Lonza) supplied with 10% FBS (Gibco, Thermo Fisher Scientific), 1% penicillin/streptomycin (Gibco, Thermo Fisher Scientific), 1 × MEM Non-essential Amino Acid Solution (Sigma-Aldrich), and 1% L-glutamine (GE healthcare). When passaging, cells were washed with DPBS (Gibco, Thermo Fisher Scientific) and detached using 0.25% trypsin (Gibco, Thermo Fisher Scientific) supplemented with 0.5 mM EDTA (Invitrogen, Thermo Fisher Scientific).

### Cell Proliferation Staining and Mammosphere Formation Assay

Cells were stained with PKH26 Red Fluorescent Cell Linker Kit (Sigma-Aldrich), according to the manufacturer’s instructions with some modifications. Briefly, cells were trypsinized and washed once with serum-free media. 1 × 10^6^ cells were resuspended in 1 ml Diluent C and then mixed with 1 ml Diluent C containing 2 μM PKH26. After 3 min in room temperature the staining was inactivated by addition of 2 ml FBS (Gibco, Thermo Fisher Scientific) for 1 min in room temperature. Cells were then centrifuged for 10 min at 300 g and washed 3 times with 5 ml complete medium. For each wash, cell suspension was transferred to a clean tube. Finally, cells were resuspended in 1 ml DPBS.

For non-adherent mammosphere formation assay, single cells were generated using a 25G needle (HSW FINE-JECT). 90,000 cells were added to T175 flasks (TPP) pre-coated with 1.2% poly(2-hydroxyethyl methacrylate) (Sigma-Aldrich) in 95% ethanol (Apoteket) using 30 ml phenol-red free DMEM/F-12 medium supplied with 2% B-27 supplement (both Gibco, Thermo Fisher Scientific), 1% penicillin/streptomycin and 20 ng/ml epidermal growth factor (Corning).

After 120 h, spheres were carefully collected with a pipette and centrifuged for 3 min at 10 g to include spheres, but avoid single cells. The supernatant was discarded leaving 2 ml of media in the bottom of the tube. Mammospheres from 6 T175 flasks were pooled and then centrifuged for 5 min at 120 g. The cells were resuspended in 0.25% trypsin followed by 3 min incubation at 37°C. A single-cell suspension was obtained using a 25G needle and trypsin was inactivated using complete medium. Cells were centrifuged for 5 min at 580 g, washed once with DPBS, centrifuged for 5 min at 300 g and resuspended in DPBS supplied with 2% bovine serum albumin (Sigma-Aldrich). Finally, cells were filtered through a 70 μm cell strainer into a FACS tube (both Falcon, Corning) and kept on ice until sorting.

### Cell Cycle Staining

To sort cells in the G1 cell-cycle phase, cells were trypsinized and 1 × 10^6^ cells/ml were resuspended in Hanks’ balanced salt solution (Gibco, Thermo Fisher Scientific) and stained for nuclear DNA using Vybrant DyeCycle Violet stain (Invitrogen, Thermo Fisher Scientific) using a final concentration of 5 μM for 30 min at 37°C. After staining, cells were resuspended in Hanks’ balanced salt solution and filtered through a 70 μm cell strainer into a FACS tube. Cells were kept on ice until sorting.

### Single-Cell Sorting

Single cells were sorted using a BD FACSAria II instrument and the FACSDiva software (both BD Biosciences). Single cells with high or low PKH26 intensity or with low intensity of Vybrant DyeCycle Violet were sorted into 96-well PCR plates (Applied Biosystems, Thermo Fisher Scientific) containing 5 μl lysis buffer containing 1 μg/μl BSA supplied in 2.5% glycerol (Thermo Scientific, Thermo Fisher Scientific) and 0.2% Triton X-100 (Sigma-Aldrich). Three wells were kept with only lysis buffer as cell-free controls. After sorting, plates were immediately frozen on dry ice and stored in -80°C.

### RNA Sequencing

The Smart-seq2 protocol was used to generate a sequencing library (Picelli et al., 2014). Reverse transcription was performed directly on the 96-well plates with collected cells. First, 1 μM of an adapter sequence-containing oligo-dT30VN (5′-AAGCAGTGGTATCAACGCAGAGTACT30VN-3′), 1 mM dNTP (both Sigma-Aldrich), and 0.04 μl of a 1:100,000 dilution of ERCC RNA Spike-In Mix 1 (Ambion, Thermo Fisher Scientific) were added to the sample followed by incubation at 72°C for 3 min and cooling to 4°C. Next, 1 × first-strand buffer (50 mM Tris–HCl pH 8.3, 75 mM KCl, and 3 mM MgCl_2_), 5 mM dithiothreitol (both Invitrogen, Thermo Fisher Scientific), 10 mM MgCl_2_ (Ambion, Thermo Fisher Scientific), 1 M betaine (Sigma-Aldrich), 0.6 μM adapter sequence-containing template switching oligonucleotides (5′-AAGCAGTGGTATCAACGCAGAGTACATrGrG+G-3′ with rG = riboguanosine and +G = locked nucleic acid modified guanosine, Eurogentec), 15 U RNaseOUT and 150 U SuperScript II (both Invitrogen, Thermo Fisher Scientific) were added to a final volume of 15 μl. Reverse transcription was performed in a PTC-200 instrument (MJ Research) at 42°C for 90 min and 70°C for 15 min. cDNA was stored at -20°C. Concentrations indicated refer to the final reverse transcription reaction.

Preamplification was performed in a 50 μL reaction containing 1 × KAPA Hifi HotStart ReadyMix (KAPA Biosystems), 0.1 μM primer (5′-AAGCAGTGGTATCAACGC AGAGT-3′; Sigma-Aldrich) and 7.5 μl cDNA. Preamplification was performed in a PTC-200 instrument at 98°C for 3 min followed by 23 cycles of amplification at 98°C for 20 s, 67°C for 15 s, and 72°C for 6 min and a final additional incubation at 72°C for 5 min. Samples were transferred from 72°C directly to dry ice and stored at -20°C.

Concentration and quality of preamplified cDNA were assessed with Agilent High Sensitivity DNA Kit on a 2100 Bioanalyzer Instrument (Agilent Technologies). To determine the concentration range of the samples, 33 randomly selected samples were analyzed. Based on these concentrations, 2 μl preamplified cDNA was diluted 1:100 in RNase/DNase-free water (Invitrogen, Thermo Fisher Scientific) and 2 μl diluted sample was used for tagmentation and indexing, in order to not exceed 100 pg cDNA. In total, 52 each of high and low intensity of PKH26 and 31 G1 cells, were further processed. Tagmentation and indexing were performed using Nextera XT DNA Library Preparation Kit and Nextera XT Index Kit v2 (Illumina). To each sample, 10 μl Tagment DNA Buffer and 5 μl Amplicon Tagment Mix was added in a total volume of 20 μl and tagmentation was performed in a PTC-200 instrument at 55°C for 5 min followed by cooling to 10°C. 5 μl Neutralize Tagment Buffer was added followed by centrifugation for 1 min at 1100 rpm and 5 min incubation at room temperature. A mixture of 15 μl Nextera PCR Master Mix and 5 μl of each index 1 (i7) and index 2 (i5) adapters was added to a total volume of 50 μl. Library amplification was performed in a PTC-200 instrument at 72°C for 3 min, 95°C for 30 s followed by 16 cycles of amplification at 95°C for 10 s, 55°C for 30 s, and 72°C for 30 s and a final additional incubation at 72°C for 5 min followed by cooling to 10°C.

Amplified samples were purified using Agencourt AMPure XP beads (BD Biosciences). All sample volume was added to 30 μl beads generating a beads-to-sample ratio of 0.6 and suspension was mixed by pipetting. DNA binding to beads was performed for 5 min at room temperature followed by 5 min incubation on a magnet (DynaMag, Thermo Fisher Scientific). Supernatant was discarded and beads washed twice with 200 μl 80% ethanol. Beads were left to dry and sample was eluted with 17.5 μl RNase/DNase-free water for 2 min followed by 2 min incubation on magnet before eluted sample was retrieved.

The mass concentration of each sample was analyzed using Qubit dsDNA High Sensitivity Assay Kit (Invitrogen, Thermo Fisher Scientific). To assess the quality and molarity, 35 selected samples were analyzed with Agilent High Sensitivity DNA Kit. The average mean size was used to calculate the molar concentration of each sample. Samples with lower concentration than 5 nM were excluded and remaining samples were diluted to 5 nM and pooled. The library pool was purified once more using Agencourt AMPure XP beads with a beads-to-sample ratio of 0.6 and diluted to 3 nM.

Sequencing of pooled single-cell libraries were performed by Genomics Core facility at the University of Gothenburg on a NextSeq 500 instrument (Illumina) using a Nextseq500 Kit High Output V2 with paired-end sequencing and a read length of 2 × 150 bp.

### Single-Cell Transcriptome Data Analysis

Reads were aligned to the human genome (hg19), with ERCC spike-in sequences included, using STAR (Dobin et al., 2012) with splice junctions supplied from the GENCODE (Harrow et al., 2012) V17 annotation. Gene expression levels were assessed by binning reads to genes using HTseq (Anders et al., 2015) with the options “-s no” and “-m intersection-strict.” The quality of each sample was assessed using FastQC (Andrews, 2010). In total, four samples failed either in the sequencing process or during quality assessment.

Genes were filtered based on variability in comparison to a noise level estimated from the ERCC spike-ins as described (Brennecke et al., 2013), utilizing the dependence of technical noise on the average read count and fitting a model to the ERCC spike-ins. All genes with squared coefficient of variation above the noise level were selected for further analyses. An additional filtering step was included only selecting genes expressed in at least 5% of all cells.

Cells were clustered using a modified version of the RaceID algorithm (Grun et al., 2015). Shortly, read counts were normalized to the median transcript number across cells. Cells were clustered using *k*-means on the modified Pearson correlation matrix with Euclidian metric and data was visualized using *t*-distributed stochastic neighbor embedding. Cluster number *k* = 3 was chosen and the stability of the clusters were assessed with Jaccard’s similarity. Only genes selected using the filtering method described above were included in the clustering, without any additional filtering. The RaceID algorithm also includes an outlier detection method which was omitted.

Pseudotemporal ordering of cells was performed using the TSCAN algorithm (Ji and Ji, 2016), which first groups cells into clusters and then orders cells along a pseudotemporal path using a minimum spanning tree approach. For this method, all genes were used and filtered according to the default filtering in TSCAN. Normalized values received from the RaceID algorithm were used.

Differentially expressed genes between clusters were identified using the SCDE algorithm (Kharchenko et al., 2014). The raw data matrix, including all genes, was used for this algorithm. Adjusted *p*-values were calculated from the cZ-values received as output from the algorithm. Genes with an adjusted *p*-value less than 0.05 were considered significant.

Gene set analysis was performed using the molecular signatures database (MSigDB) (Subramanian et al., 2005). The gene lists obtained from differential expression analysis was compared with the Hallmark, Reactome and KEGG gene sets as well as transcription factor targets. Top 100 significantly enriched gene sets (cutoff of FDR *q* = 0.05) were identified.

Survival data was assessed using the Kaplan-Meier plotter tool for breast cancer^1^ which is based on available microarray data (Gyorffy et al., 2010). Relapse-free survival was assessed using JetSet to select the optimal probe (Li et al., 2011) and auto-selection of best cutoff for dividing the patients into low and high expression of each gene. The analysis was performed separately on patients belonging to different intrinsic subgroups. Furthermore, ERα positive and negative breast cancer patients were analyzed as well as ERα negative patients with or without systemic treatment. Apart from this, no other selections of patients were done. To distinguish significantly altered survival, *p*-values were adjusted for multiple testing using false discovery rate (Benjamini-Hochberg procedure). The *p*-value adjustment was performed separately for each subgroup of patients including *p*-values for all analyzed genes.

## Results

### Enrichment of Breast Cancer Stem Cells and Identification of Biologically Variable Genes

**FIGURE 1:**
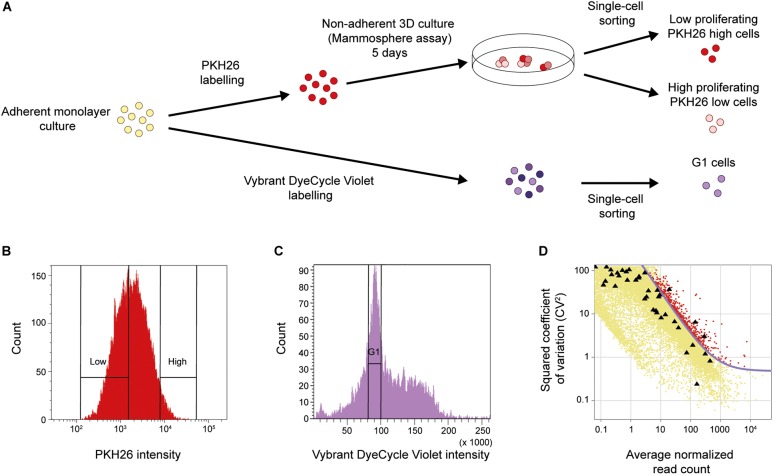
Functional CSC enrichment and identification of biologically variable genes. **(A)** Cellular assays. Upper panel: MDA-MB-231 cells were stained with the PKH26 membrane dye to assess proliferation and then functionally enriched for resistance to anoikis using the non-adherent mammosphere assay. Low and high proliferating single cells were collected with fluorescence-activated cell sorting (FACS) using the intensity of the incorporated PKH26 dye (PKH26 high cells and PKH26 low cells, respectively). Lower panel: MDA-MB-231 cells were stained with the DNA-binding dye Vybrant DyeCycle Violet to assess cell-cycle phase. Single cells were then directly collected from the G1 phase using the intensity of the incorporated dye (G1 cells). **(B)** FACS of PKH26-stained mammosphere cells. The cells were sorted into two groups based on their PKH26 intensity, The PKH26 high and PKH26 low cells constituted 3 and 40% of the total cell population, respectively. The rationale behind this gating strategy was to enrich for the least proliferating cells (PKH26 high cells), while the proliferating cell population (PKH26 low cells) should reflect a wider variety of dividing cells. **(C)** FACS of Vybrant DyeCycle Violet-stained cells. Cells from the G1 phase were sorted based on low dye intensity. **(D)** Identification of biologically variable genes. The plot shows the level of variation (CV^2^) against the average expression level of each gene. External ERCC spike-in controls (black triangles) were added to each cell to assess the technical noise. The noise level (purple curve) was fitted from the ERCC controls. Genes with a CV^2^ above the noise level and expressed in 5% of the samples (576 genes in total, red data points) were used for downstream analysis.

It has been shown that low proliferation (Dembinski and Krauss, 2009) and the ability to form spheres under non-adherent conditions (Dontu et al., 2003) are traits linked to the CSC populations. To functionally enrich for breast cancer cells with these characteristics, we applied the mammosphere assay in combination with the label-retention PKH26 assay on breast cancer cell line MDA-MB-231, as shown in Figure 1A. Cells were first labeled with the membrane dye PKH26 before being cultured as mammospheres for 5 days. Low (PKH26 high cells) and high (PKH26 low cells) proliferating single cells dissociated from mammospheres were collected (Figure 1B). As control, normal adherent monolayer cells in the G1 cell cycle phase were collected using the Vybrant DyeCycle Violet DNA-binding dye (Figure 1A,C). We performed whole transcriptomic analysis at single-cell level applying the Smart-Seq2 protocol (Picelli et al., 2014). After sequencing and sample quality control we used 46 low proliferating mammosphere cells (PKH26 high cells), 45 high proliferating mammosphere cells (PKH26 low cells) and 30 cells in G1 cell cycle phase cultured in adherent monolayer conditions (G1 cells) for further analyses.

Next, we identified relevant genes with biological variation above the technical noise (Brennecke et al., 2013). Briefly, the technical noise level was assessed from the variation and expression of ERCC spike-in controls which were added to each sample before library preparation (Figure 1D). In total, 576 genes were identified with a variability above this technical noise level and expressed in at least 5% of all cells.

### Identification of a Cancer Stem Cell Subpopulation

**FIGURE 2:**
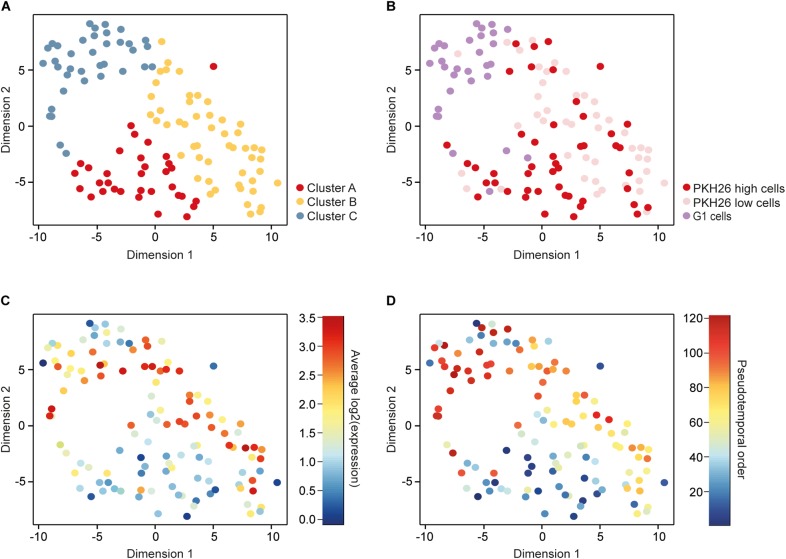
Identification and characterization of subpopulations. **(A)** A *t*-distributed stochastic neighbor embedding (*t*-SNE) plot visualizing three clusters of cells identified with *k*-means based on their gene expression profiles. **(B)** The same *t*-SNE plot as in **A**, with cells colored according to their cellular phenotype, including low (PKH26 high cells) and high (PKH26 low cells) proliferating mammosphere cells as well as adherent 2D cells in the G1 cell-cycle phase (G1 cells). **(C)** The same *t*-SNE plot as in **A**, with cells colored according to their average expression of cell-cycle related genes, represented by the genes among the 576 remaining after filtering that were included in the Reactome cell cycle gene set from the molecular signatures database (343 genes in total). **(D)** The same *t*-SNE plot as in **A**, with cells colored according to their pseudotemporal ordering from 1 to 121.

To identify subpopulations of cells we applied parts of the RaceID algorithm (Grun et al., 2015), which groups cells using *k*-means clustering and visualizes the obtained subpopulations with *t*-distributed stochastic neighbor embedding (*t*-SNE). This approach identified three cell clusters (Figure 2A).

To compare the clustering based on gene expression to the functional properties assessed based on the culture and staining procedures, we overlaid the cellular phenotype in the *t*-SNE plot (Figure 2B). Cluster A mainly consisted of PKH26 high cells (76%), 69% of the cluster B cells were PKH26 low cells, while 73% of the cluster C cells were G1 cells. These results show that the enriched cellular phenotypes were also reflected at gene expression level. To further confirm this relationship, we overlaid the mean expression of cell-cycle related genes in the *t*-SNE plot (Figure 2C). High proliferating (PKH26 low cells) and G1 cells were the cells that expressed cell-cycle related genes to highest extent.

To define the gradual transition of the cells between different subpopulations we constructed a pseudotemporal ordering of all cells using the TSCAN algorithm (Ji and Ji, 2016). This algorithm orders cells depending on their gradual changes in the transcriptome profile. The pseudotemporal cell order was visualized in the *t*-SNE plot with the previously identified clusters (Figure 2D). Early ordered cells were more prone to belong to cluster A whereas cells toward the end mainly belonged to cluster C. Altogether, these data show that cluster A is the most CSC-like subpopulation.

### Subpopulation-Specific Gene Expression Profiles

To characterize the three identified subpopulations we defined differentially expressed genes using the SCDE algorithm (Kharchenko et al., 2014). Gene lists for all pair-wise comparisons are shown in Supplementary Table S1. The regulated genes were compared with the Hallmark, Reactome and KEGG gene sets in the Molecular Signatures Database (Supplementary Table S2; Subramanian et al., 2005). Furthermore, over-representation of targets for transcription factors was identified using the same database (Supplementary Table S2). Another difference between the subpopulations was that the relative amount of total transcripts was higher in cluster C compared to clusters A and B (Supplementary Figure S1A). The relative amount of total transcripts also increased toward the end of the pseudotemporal ordering (Supplementary Figure S1B).

**FIGURE 3:**
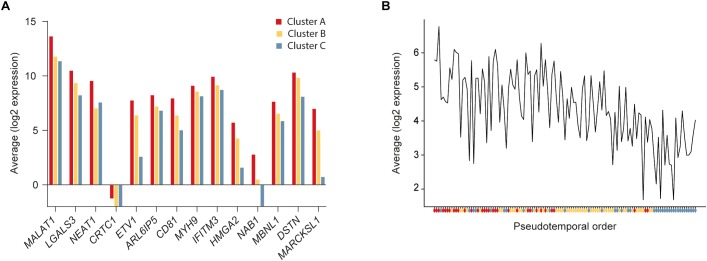
Expression of cancer stem cell (CSC) upregulated genes. **(A)** Average expression of significantly upregulated genes in cluster A compared to clusters B and C. **(B)** Average expression of the 14 upregulated genes in cluster A compared to clusters B and C along the pseudotemporal ordering. The colored dots below the x-axis represent the cluster each cell belongs to.

To further define the CSC-like cells in cluster A we identified 14 genes that were significantly upregulated in this cluster compared to the other two clusters (Table 1). The average expression of each gene in the three clusters is illustrated in Figure 3A and the average expression of all 14 genes along the pseudotemporal ordering is shown in Figure 3B. To determine the association between the 14 genes and clinical survival data for ERα positive and negative tumors we used a Kaplan-Meier analysis tool based on a collection of publicly available data (Gyorffy et al., 2010; Table 1 and Supplementary Figure S2). Furthermore, we performed the same survival analyses in patients with tumors belonging to different intrinsic subgroups (basal-like, luminal A, luminal B and HER2-enriched) as well as in patients with ERα negative tumors with or without systemic treatment (Supplementary Table S3). The data showed variable results among different genes and patient groups but three genes, *LGALS3*, *MYH9*, and *DSTN*, were significantly related to worse survival in ERα negative tumors.

**Table 1:** Genes upregulated in cluster A.

Gene name	Log2 fold change^a^	Adj. *p*-value^b^	Survival data^c^	Function^d^
*MALAT1 (non-coding)*	0.95	4.8 × 10^-9^	ERα neg. – ERα pos. –	mRNA regulation of genes connected to cell cycle regulation and cell migration
*LGALS3*	1.4	3.8 × 10^-6^	ERα neg. ↓ ERα pos. ↑	Involved in apoptosis and cell adhesion
*NEAT1 (non-coding)*	1.9	5.3 × 10^-6^	ERα neg. – ERα pos. –	Paraspeckle formation, mRNA regulation
*CRTC1*	4.1	3.0 × 10^-4^	ERα neg. ↑ ERα pos. –	Transcriptional coactivator of CREB1, involved in several pathways
*ETV1*	1.5	2.1 × 10^-3^	ERα neg. ↑ ERα pos. –	Transcription factor of the ETS family, involved in many biological processes
*ARL6IP5*	1.3	2.7 × 10^-3^	ERα neg. – ERα pos. ↑	Expression affected by vitamin A. May be associated with the cytoskeleton and glutamate transport
*CD81*	1.2	9.0 × 10^-3^	ERα neg. – ERα pos. –	Possibly involved in muscle cell fusion and signal transduction, might be connected to cell growth
*MYH9*	1.1	9.5 × 10^-3^	ERα neg. ↓ ERα pos. –	Cytokinesis, cell motility and maintenance of cell shape
*IFITM3*	0.91	1.3 × 10^-2^	ERα neg. – ERα pos. ↑	Interferon-induced membrane protein. Inhibits entry of viruses
*HMGA2*	1.2	1.5 × 10^-2^	ERα neg. – ERα pos. –	Transcriptional regulation, involved in cell cycle regulation
*NAB1*	2.2	2.2 × 10^-2^	ERα neg. – ERα pos. ↑	Transcriptional repressor for zinc finger transcription factors EGR1 and EGR2
*MBNL1*	1.1	2.2 × 10^-2^	ERα neg. – ERα pos. ↑	Alternative splicing
*DSTN*	1.1	2.5 × 10^-2^	ERα neg. ↓ ERα pos. –	Actin depolymerisation
*MARCKSL1*	1.5	2.7 × 10^-2^	ERα neg. ↑ ERα pos. –	Cell movement and migration


**^*a*^Upregulation, comparing cluster A to both other clusters. ^*b*^*p*-value adjusted for multiple testing by false discovery rate. ^*c*^Correlation of gene expression to relapse-free survival assessed by Kaplan-Meier analysis using http://kmplot.com. Significantly (FDR < 0.05) better (↑) or worse (↓) survival indicated for ERα negative and positive breast cancer. ^*d*^Functions of gene and/or encoded protein. Information from NCBI (NCBI Resource Coordinators, 2016) and/or The UniProt Consortium (2017).**

## Discussion

Breast cancer tumors are heterogeneous and harbor CSCs. Several pathways related to self-renewal are associated to breast CSCs, including the Wnt, Notch, and Hedgehog pathways (Liu et al., 2006; Harrison et al., 2010; Khramtsov et al., 2010). These signaling pathways are also connected to each other, indicating a complex system for CSC maintenance (Takebe et al., 2011). There have been attempts of targeting the CSC population through these pathways but this is associated with several challenges (Takebe et al., 2015) and no such therapy is in clinical use.

To further characterize breast CSCs we performed single-cell RNA sequencing on cellular populations functionally enriched for different CSC properties. The mammosphere assay enriches for cells that are able to self-renew and differentiate and the intensity of the PKH26 dye allows cells to be selected based on their proliferation rate (Pece et al., 2010). However, the mammosphere assay has been shown to select for a larger cell population than the CSCs only (Pastrana et al., 2011) and PKH26 is a membrane dye whose intensity readout could be affected by other cellular properties than cell division, such as cell size (Dolatabadi et al., 2017). For comparison, we included cells cultured under adherent monolayer conditions, selected from the G1 cell cycle phase only to minimize the effect of cell cycle specific gene expression (Whitfield et al., 2002). Our three single-cell defined subpopulations overlapped to a large extent with our three predefined groups, i.e., PKH26 high, PKH26 low, and G1 cells. Gene expression analysis showed proliferation to be a strong dividing factor between clusters and the cell group showing a decrease in expression of cell-cycle related genes also showed an over-representation of cells with high PKH26 intensity. These data support the accuracy of PKH26 in separating cells based on proliferation rate. In order to study the differentiation process, we ordered the cells along a pseudotemporal path. The pseudotemporal ordering fitted well with the clustering, indicating a gradual transition of cells between different states along the different subpopulations, proposedly from a more CSC-like state to a more differentiated state.

To determine the cellular functions of identified cell clusters we defined enriched gene sets (Supplementary Table S2). As expected, when comparing cluster A with cluster B this analysis generated overrepresentation of gene sets related to cell cycle, confirming the use of label-retention PKH26 dye assay. Also, enriched transcription factor sequence motifs included several targets of E2Fs, known cell-cycle regulators (Morgan, 2007) as well as NFY, shown to interact with E2Fs but also involved in stem cell self-renewal (Zhu et al., 2004, 2005; Blum et al., 2009). When comparing cluster B to cluster C, overrepresented gene sets included several processes related to CSCs, such as epithelial to mesenchymal transition (Mani et al., 2008; Morel et al., 2008), hypoxia (Schwab et al., 2012; Harrison et al., 2013) and apoptosis (Madjd et al., 2009; Kruyt and Schuringa, 2010). Also, gene sets related to protein and RNA metabolism were upregulated. Among the transcription factor targets, CREB and ATF were highly represented, which are involved in diverse physiological processes, including proliferation, survival (Mayr and Montminy, 2001) and differentiation (Masson et al., 1993; Kingsley-Kallesen et al., 1999). Finally, the cellular functions describing the differences between clusters A and C reflected a combination of functions describing differences of the other two cluster comparisons, including cell-cycle regulation, stem-cell properties and differentiation. Additionally, among transcription factor targets, AP1 was enriched, a transcription factor complex consisting of members of the JUN, FOS and ATF families that has been found to be connected to proliferation, invasion and apoptosis (Chen et al., 1996; Eferl and Wagner, 2003; Milde-Langosch et al., 2004). Global changes in RNA and protein have been seen when comparing stem cells to more differentiated cell types. Our results showed an increase in total amount of transcripts in cluster C, in line with earlier studies reporting decreased amount of total RNA in stem cells (Sampath et al., 2008; Sanchez et al., 2016).

We propose that cluster A contains the most CSC-like cells and that, for this cluster, upregulated genes are potential CSC biomarkers (Table 1). Among these genes, two were non-coding, *MALAT1* and *NEAT1*, of which both are connected to CSC properties in several cancer types, including breast cancer (Arun et al., 2016; Jen et al., 2017; Li et al., 2017). The coding genes are also related to CSC properties but also other cellular functions. For example, non-muscle myosin IIA, encoded by *MYH9*, is involved in cell motility and has been shown to be involved in migration of MDA-MB-231 cells (Betapudi et al., 2006; Dulyaninova et al., 2007). MYH9 also interacts with Galectin-3, encoded by *LGALS3*, in bone metastasis (Nakajima et al., 2016). Galectin-3 is connected to several tumor properties (Newlaczyl and Yu, 2011), including chemoresistance and stem cell properties in breast cancer (Newlaczyl and Yu, 2011; Guha et al., 2014). Furthermore, *HMGA2* is involved in metastasis through epithelial to mesenchymal transition (Thuault et al., 2006; Morishita et al., 2013). We also determined the connection between the expressions of these genes to clinical outcome using publicly available data. The varying relationships between gene and patient selection was not surprising, since it is well known that breast cancer subgroups, like ERα positive and negative breast cancer, often display divergent expression pattern (Reis-Filho and Pusztai, 2011). The three genes; *LGALS3*, *MYH9*, and *DSTN*, that were significantly associated with worse relapse-free survival in ERα negative breast cancers are of extra interest for future studies. Two of those genes, *LGALS3* and *DSTN*, were significantly related to worse survival specifically in patients that had not received systemic treatment, whereas the opposite were true for *MYH9*. Additionally, *NEAT1* was connected to worse survival in systemically treated ERα negative patients, although the analysis was performed in a smaller patient cohort (Supplementary Table S3). Looking into the intrinsic subgroups, two of the above-mentioned genes, *LGALS3* and *DSTN*, were significantly associated to worse survival in several subgroups including the basal group, in which also *NAB1* was connected to worse survival.

## Conclusion

In conclusion, we have identified potential breast cancer biomarkers related to CSC properties, especially associated with ERα negative breast cancer, using functional cellular assays combined with single-cell gene expression profiling. Further experimental validation, using more cell lines as well as other model systems, is needed to confirm their cellular functions and potential clinical use.

## Data Availability Statement

RNA sequencing data have been deposited in NCBI’s Gene Expression Omnibus (Edgar et al., 2002) database with accession number GSE124989.

## Author Contributions

EJ and AS conceived the study. EJ, EP, and TK performed the experiments. EJ, SG, JK, EL, GL, and AS analyzed the data. EJ and AS drafted the manuscript. All authors read and approved the final manuscript.

## Conflict of Interest Statement

AS declares stock ownership in TATAA Biocenter. The remaining authors declare that the research was conducted in the absence of any commercial or financial relationships that could be construed as a potential conflict of interest.

## Footnotes

1 http://kmplot.com
