# Erratum: Identification of Breast Cancer Stem Cell Related Genes Using Functional Cellular Assays Combined With Single-Cell RNA Sequencing in MDA-MB-231 Cells

**DOI:** 10.3389/fgene.2020.00084

**Published:** 2020-02-05

**Authors:** Frontiers Production Office

**Affiliations:** Frontiers Media SA, Lausanne, Switzerland

**Keywords:** breast cancer, cancer stem cell, cell proliferation assay, mammosphere assay, single-cell analysis, single-cell RNA sequencing

The Data Availability Statement was missing in the original article. A correction has therefore been made, and the statement should read:

“RNA sequencing data have been deposited in NCBI's Gene Expression Omnibus (Edgar et al., 2002) database with accession number GSE124989.”

The original version of this article has been updated.
