# Supplementary material for: Identification of Breast Cancer Stem Cell Related Genes Using Functional Cellular Assays Combined With Single-Cell RNA Sequencing in MDA-MB-231 Cells
Source: Front Genet. 2019 May 22;10:500. doi: 10.3389/fgene.2019.00500 (PMC6541172; doi:10.3389/fgene.2019.00500)
Supplement: Supplementary file 3 [file Table_3.DOCX]

Table S3. Genes upregulated in cluster A.

|  |  | **Intrinsic subtype^b^** | | | | **ERα status^b^** | | **ERα-, systemic treatment (yes/no)^b^** | |
| --- | --- | --- | --- | --- | --- | --- | --- | --- | --- |
| **Gene name** | **Probe set ID^a^** | **Basal** | **Lum A** | **Lum B** | **HER2+** | **ERα+** | **ERα-** | **Yes** | **No** |
| *MALAT1* | 224559_at | ↑ | - | - | - | - | - | - | - |
| *LGALS3* | 208949_s_at | ↓ | - | ↓ | ↓ | ↓ | ↓ | - | ↓ |
| *NEAT1* | 224565_at | ↑ | ↑ | ↑ | - | - | - | ↓ | - |
| *CRTC1* | 213091_at | ↑ | ↑ | ↑ | - | - | ↑ | - | ↑ |
| *ETV1* | 221910_at | ↑ | ↑ | ↑ | ↑ | - | ↑ | - | - |
| *ARL6IP5* | 200761_s_at | ↑ | ↑ | - | - | ↑ | - | - | - |
| *CD81* | 200675_at | ↑ | - | - | - | - | - | - | - |
| *MYH9* | 211926_s_at | - | ↑ | - | - | - | ↓ | ↓ | - |
| *IFITM3* | 212203_x_at | - | ↑ | - | - | ↑ | - | - | - |
| *HMGA2* | 1558683_a_at | ↑ | ↑ | ↑ | ~~-~~ | - | - | - | - |
| *NAB1* | 209272_at | ↓ | ↑ | - | ↓ | ↑ | - | - | - |
| *MBNL1* | 201153_s_at | ↑ | ↑ | - | - | ↑ | - | - | - |
| *DSTN* | 201022_s_at | ↓ | ↓ | ↓ | - | - | ↓ | - | ↓ |
| *MARCKSL1* | 200644_at | ↑ | ~~-~~ | ↓ | - | - | ↑ | - | - |

# ^a^ Probe set used for the analysis. ^b^ Correlation of gene expression to relapse-free survival assessed by Kaplan-Meier analysis using http://kmplot.com. Significantly (FDR < 0.05) better (↑) or worse (↓) survival indicated. Color of arrow indicates patient number (reliability of analysis) as described by the Kaplan_meier Plotter tool; green: more than 500 samples (highly reliable analysis), blue: 500-200 samples (neutral), yellow: 200-50 samples (preliminary analysis).
