# Supplementary figures and images for: Identification of Breast Cancer Stem Cell Related Genes Using Functional Cellular Assays Combined With Single-Cell RNA Sequencing in MDA-MB-231 Cells
Source: Front Genet. 2019 May 22;10:500. doi: 10.3389/fgene.2019.00500 (PMC6541172; doi:10.3389/fgene.2019.00500)

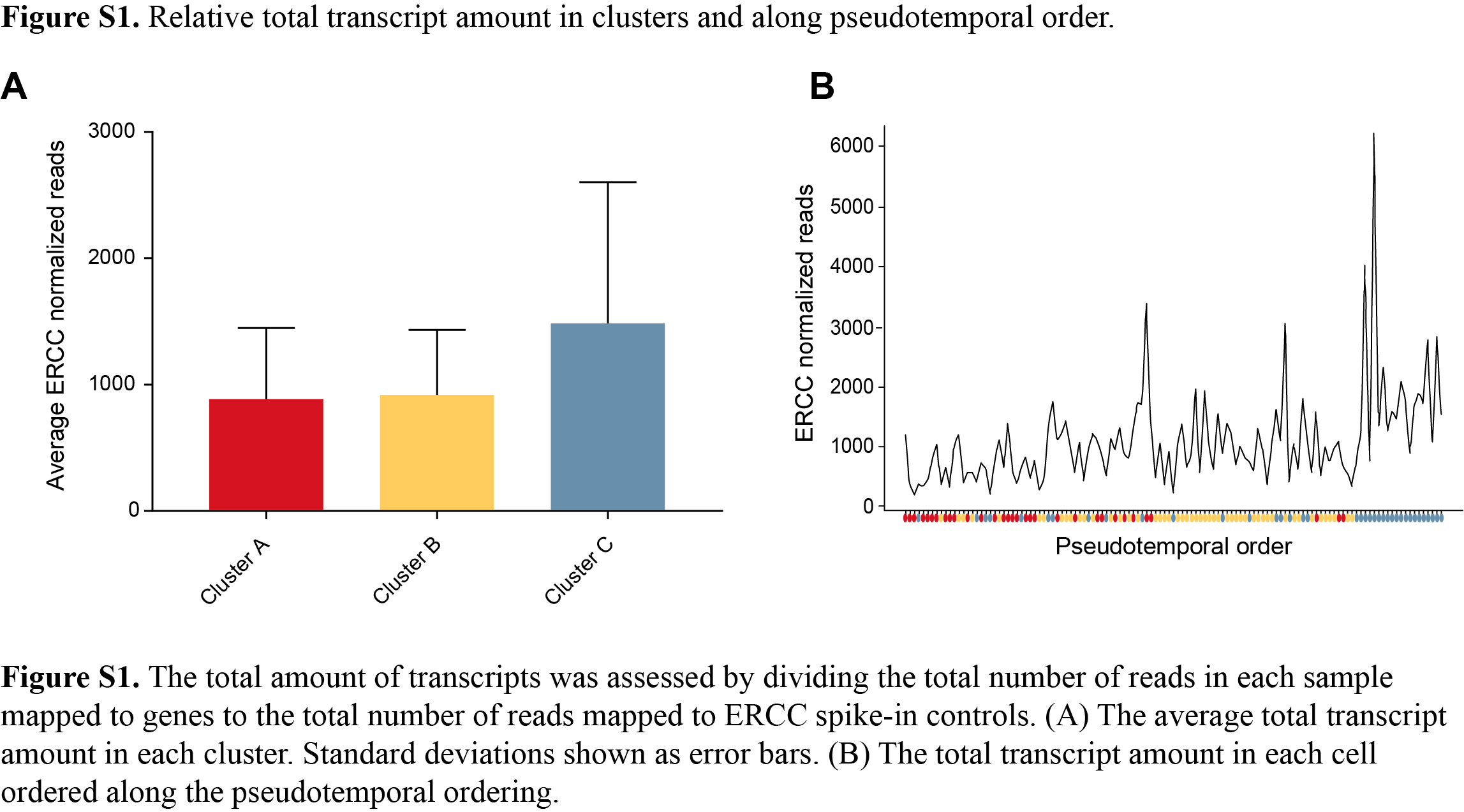

Supplement: Supplementary file 4 [file Image_1.png]
